# Mentorship—A critical metric for career development and advancing global health

**DOI:** 10.1371/journal.pgph.0005247

**Published:** 2025-10-15

**Authors:** Jessica E. Haberer, Pauline Byakika-Kibwika, Monica Gandhi, Elizabeth A. Bukusi, Moses Kamya, Yap Boum II

**Affiliations:** 1 Center for Global Health and Department of Medicine, Massachusetts General Hospital, Boston, Massachusetts, United States of America; 2 Department of Medicine, Harvard Medical School, Boston, Massachusetts, United States of America; 3 Mbarara University of Science and Technology, Mbarara, Uganda; 4 Division of HIV, Infectious Diseases, and Global Medicine, University of California San Francisco, San Francisco, California, United States of America; 5 Center for Microbiology Research, Kenya Medical Research Institute, Nairobi, Kenya; 6 Departments of Obstetrics and Gynecology and Global Health, University of Washington, Seattle, Washington, United States of America; 7 Department of Medicine, Makerere University, Kampala, Uganda; 8 Infectious Diseases Research Collaboration, Kampala, Uganda; 9 Incident Management Support Team, Africa Centers for Disease Control and Prevention, Kinshasa, Democratic Republic of Congo; 10 Faculty of Medicine and Biomedical Sciences, University of Yaoundé I, Yaoundé, Cameroon; Worcester Polytechnic Institute, UNITED STATES OF AMERICA

## Abstract

Mentorship plays a critical role in promoting career development and generating impactful research and programs, yet it is typically considered an altruistic endeavor and a luxury. Mentorship programs are largely unfunded and unsupported, particularly in the Global South. However, they are important in global health given the high need for mentors in many settings as well as the cross-cultural complexities and power dynamics inherent in the field. Mentor training programs are becoming increasingly available and have been shown to increase traditional metrics of impact—namely, manuscripts, grants, and program milestones. However, the current focus on these metrics without supporting mentorship can detract from their impact, leading to quantity over quality and programs disconnected from the communities they are meant to serve. In this article, we argue that making mentorship itself a metric will facilitate the true impact we seek in global health, while simultaneously promoting equity in opportunity. We describe mechanisms to ensure quality in mentorship and highlight the importance of governmental and non-governmental agencies and philanthropy in enabling institutions to implement mentorship programs. We further describe increasing access to mentorship at scale through digital platforms like The Village, which enables and supports mentor-mentee matches. Elevating mentorship to a core performance indicator will have profound downstream effects for the practice and products of global health.

## Mentorship

The word conjures images of dedicated senior scholars nurturing eager young students through their studies or providing counsel as students contemplate training options for their career development. For many, mentorship is the key to success. Dedicated mentors embrace the vision of who their mentees wish to become. They guide them through academic programs, advise them on career decisions, and sponsor them to obtain new opportunities… sometimes for many years. The mentor-mentee relationship is founded on commitment and mutual respect that leads to career advancement for the mentee and personal and professional fulfillment for the mentor. While often the relationship does involve a pairing of senior and junior colleagues, it can also include peer mentors and teams of mentors with complementary perspectives that may evolve over time.

Unfortunately, this idyllic relationship is far too rare. Effective mentorship may arise through chance or perhaps through the support and encouragement of institutions (e.g., universities, organizations, and programs); however, it is rarely considered an essential professional activity, leading to countless missed possibilities in global health (and beyond). Mentorship is particularly important in the field of global health given the high need for mentors in much of the Global South as well as the cross-cultural complexities and power dynamics inherent in global health. This article argues that re-envisioning mentorship as a necessary metric to advance global health will facilitate its uptake and implementation within institutions globally. This approach is needed to both promote equitable opportunities as well as overcome current limitations in traditional assessments of impact. In this context, we consider mentorship as any long-term relationship in which support is provided to advance an individual’s career; it could involve students or faculty at any stage of their education and professional development. We believe these principles apply broadly across any aspect of global health from public health to the social and laboratory sciences to medicine.

### Barriers to implement mentorship

Mentorship is often seen as a luxury rather than a necessity, leaving many individuals without access to meaningful mentorship opportunities. Supervision—typically tied to a task like a manuscript or thesis—is more common. While supervision can be instrumental in achieving specific goals, it is often cursory and superficial. Many institutions globally, but particularly in the Global South, lack formal mentorship programs, and where such programs exist, mentors are scarce. They are frequently overburdened with too many mentees, insufficient training, and no protected time for meaningful, sustained engagement [1,2]. Without training, mentors often struggle to understand their roles, while mentees seldom receive guidance on how to engage effectively with their mentors. These gaps are exacerbated by the absence of mentorship as a criterion in performance and promotion [3], relegating mentorship efforts to evenings and weekends where it can feel like a burden.

In the Global South, mentorship faces additional challenges such as limited funding for capacity building, which is often controlled by external organizations with little local ownership or control [4]. A robust mentorship culture is also lacking, particularly among people early in their careers, and available mentors are few. Furthermore, negative perceptions of research and public health as career paths—viewed as less lucrative than consultancies or deprioritized in favor of classroom teaching—further discourage mentorship. Little emphasis on mentorship by governments and funders, weak collaboration among stakeholders, and the lack of regional, national, or international strategies to support mentorship exacerbate these issues. These barriers coupled with poor awareness of the value of mentorship mean that successful mentoring relationships often depend more on chance than design, resulting in lost opportunities to realize the full potential of mentorship.

### Programs to promote mentorship

This scenario is driving efforts in the global health community to promote the value of well-supported mentorship. In the Global North, institutions increasingly offer mentorship workshops, embracing the culture of long-term commitment to mentees’ careers. Protected time for mentorship is still uncommon, but “mentorship” is becoming a buzz word. Examples of such programs include the Mentoring the Mentors Program at the University of California San Francisco, the Center for the Improvement of Mentored Experiences in Research at the University of Wisconsin, and the United States National Institutes of Health (NIH) National Research Mentoring Network. Similar programs are also being developed in the Global South, although to a lesser extent. A recent systematic review identified 20 research mentorship programs in 12 African countries across the continent [5]. Partnerships and collaborations were “heavily driven” by the Global North with only two initiated with local funding. Several programs involved regional and in-country collaborations and specialized capacity-building programs (e.g., mental health) with notable benefits for skill development and career advancement.

Common curricular elements of mentorship programs are listed in Table 1 [6,7]. These programs teach fundamental skills, including the many roles mentors may play, various leadership styles to facilitate mentee growth and productivity, techniques for giving and receiving effective feedback, time management, recommendations on using emotional intelligence to elevate the best in mentees, and guidance for addressing any personal differences between mentors and mentees (e.g., gender, race, or generation). Particularly in global health, topics on cross-cultural communication and distance mentoring are available, which complement existing literature and resources on global health mentorship and education [8–10]. These elements are essential in the field of global health which inherently involves power dynamics and equity considerations. These mentorship programs also provide tools and structure (e.g., templated agendas for regular meetings with planned follow-up and individual development plans) that encourage progress through specific tasks and career advancement. Many programs additionally provide guidance to mentees to help them actively engage and effectively communicate with their mentors. High-quality mentorship utilizing these tools has been shown to yield excellent returns on investment using common metrics of success, including manuscripts, grants, and programmatic milestones [5,6,11].

**Table 1:** Sample curricula from mentoring programs.

Mentor training
• Mentor roles (e.g., coach, sponsor, advisor, guide)
• How to structure the mentor-mentee relationship
• Leadership styles, influences on mentoring, how to blend effectively
• Personality styles and relationship to mentoring
• Emotional intelligence
• Giving and receiving feedback
• Cross-cultural communication, including navigation of power dynamics
• Distance mentorship
• Mentoring across difference (e.g., gender, race, generation)
• Guidance for mentee skills (e.g., active engagement, communication style)
• How to mentor grant writing
• How to mentor manuscript writing
• Time management in mentoring
**Tools for mentorship**
• Individual development plan
• Templated mentee agenda for weekly meetings with follow-up specifications
• Time management matrix
• Personality/style surveys
• Feedback matrix

### Traditional metrics of impact and their limitations

Indeed, the products of high-quality mentorship are commonly the metrics by which much of academia, including in clinical medicine and public health, are judged. Manuscripts illustrate knowledge generated from one’s work, grants demonstrate the ability to conduct and garner funding for impactful research, and program milestones reflect improvements in health. These metrics are logical and can clearly indicate progress on investment. Bill Gates said, “I have been struck again and again by how important measurement is to improving the human condition.” Yet a key mechanism for promoting the advancement of these metrics—mentorship—is not a metric itself in most institutions.

Importantly, these other metrics have the potential to compete with mentorship and even distort the impact they are meant to measure. Time spent on mentorship is time spent away from mentors writing their own manuscripts and grants or performing other duties. The phrase “publish or perish” is well known by all. Some mentors manage to “do it all”, but most struggle and many burn out with the pressure. Intense focus on these metrics can also diminish their intended value. For example, emphasis on manuscripts can result simply in a high number of publications, not necessarily good science that leads to high-quality publications or improvements in health. Predatory journals facilitate publishing almost anything, including artificial intelligence (AI)-generated content [12,13]. The peer review process has also become overtaxed with too many manuscripts for too few experienced reviewers who are uncompensated for their time; the quality of the science therefore often depends more squarely on investigators themselves. Similarly, grants may be written simply to get more grants, producing research that is isolated in academia and disconnected from real-world impact. Program development and implementation may focus solely on achieving milestones (typically set by the Global North) without appropriate attention to communities and context [14,15].

### Proposing mentorship as a metric

Clearly, many upstream institutional and societal factors also influence the design and execution of these potentially useful metrics; mentorship is not a silver bullet. However, guidance from a dedicated mentor can play a vital role in addressing many important needs for mentees as they strive to write manuscripts and grants or achieve program milestones. Mentorship can help ensure that advancement in science, medicine, and public health are the goals that accompany the manuscripts, grants, and program implementation. The wisdom of the mentor can encourage mentees to stay focused on the content of the work, not just the quantity, and to engage respectfully with communities involved. Mentors provide critical context, guiding their mentees through the logistics of attaining these metrics while maintaining their value and impact. At the same time, mentors benefit from the questions and ideas their mentees bring to the discussions as well as the clarity that comes through the process of teaching and guiding their mentees. We propose mentorship itself as a metric of success in guiding research and programs toward optimized professional development that will then translate into desired outcomes in both education and health outcomes.

### Mentorship to address disparities between the Global South and Global North

Specific to global health, mentorship is also vital for addressing inequities in opportunities between the Global South and Global North. Mentors can help identify and elevate those who have much to contribute but lack the opportunities to do so. The focus on manuscripts, grants, and programmatic milestones can make starting a career intimidating without additional support. Metrics set by the Global North may also not be intuitive to those in the Global South, and the “rules” for obtaining grants may not be well understood without a mentor to translate them. Mentorship can also inspire mentees to advocate for their own priorities in the global agenda regardless of their geography or institution. Mentorship, in sum, can be a new metric for making a difference in global health.

### Ensuring quality in mentorship

As with other metrics, a mentorship metric could become distorted without careful attention to quality. Commonly, mentors are judged by the number and productivity of their mentees—their manuscripts, grants, and other achievements—which are vulnerable to the above-noted weaknesses. However, multiple tools involving the mentors themselves, their mentees, and their institutions (Table 2) can help avoid these challenges. Mentors can document meetings with their mentees, and a supervisor can conduct brief confidential interviews with mentees to learn if the relationship is positive and professional goals are being met. Similar assessments can be made for mentees’ engagement with their mentors. Institutions can be assessed for the availability and utilization of mentor training, and session materials can be reviewed for fidelity to evidence-based approaches and curricula. Inclusion of mentorship in performance reviews, promotions, and tenure criteria is critical to avoid sole reliance on altruism [3], and the institutional environment should be conducive for mentorship. Publicized awards for high quality mentorship can help support this culture, and leadership can prioritize mentorship in hiring practices and institutional goals to encourage the practice as a professional norm [16,17]. Mentorship should not be an unsupported mandate, but rather time should be allotted and, ideally, funding should be provided for mentorship [1,2].

**Table 2:** Metrics of high-quality mentorship, beyond traditional metrics of mentee productivity (e.g., manuscripts, grants, and programmatic milestones).

Mentors
• Time spent with mentee
• Mentor Strength of Relationship Scale [20]
• Confidential interview with mentee
**Mentees**
• Time spent with mentor
• Mentee Strength of Relationship Scale [20]
• Confidential interview with mentor
**Institutions/programs**
• Availability and utilization of mentor training
• Quality of mentor training (evidence-based, audited observation)
• Inclusion of mentorship in promotion criteria
• Supportive environment for mentorship (time, funding)
• Awards for high quality mentorship
• Inclusion of mentorship expertise and activities in hiring and performance reviews

Important lessons can be learned about mentorship metrics from other professions. For example, in the technology industry, promotion dossiers require individuals to describe how they help their mentees grow and how they have benefitted from their mentoring relationships [18]. In corporate programs (e.g., in consulting firms), some firms have developed “mentorship indices” that describe the frequency and depth of mentoring relationships through institution-wide surveys and shared feedback to promote accountability and best practices [19].

### The role of funders in promoting mentorship

Governmental and non-governmental agencies and philanthropy have great potential to promote high-quality mentorship that can have significant global health impact. Several programs already sponsor training initiatives (e.g., NIH’s D43 International Research Training Grants and R25 Educational Project Grants). The inclusion and role of mentorship training in these initiatives, however, is uneven. Adding an expectation of mentor training and support to these training programs, as was recently done for NIH T32 postdoctoral training programs, would add little cost and could have dramatic returns. The NIH has also supported K24 Mid-Career Investigator Awards that provide protected time for mentoring, and the European Union and South Africa Medical Research Council offer similar awards. A single well-supported mentor has the potential to enhance the careers of dozens of mentees. Expansion of these efforts could yield immense returns on investment for science and public health and may be even more important in the currently changing landscape of foreign aid and investment.

### Equity in mentorship opportunities

Making high-quality mentorship a supported metric will advance its prioritization and help it grow more rapidly where it is needed most, greatly improving equity in global health. Currently, mentorship is largely the purview of well-funded institutions, as evidenced by the current disproportionate availability of mentorship training in the Global North compared to the Global South. Opportunities beget opportunities, but only for those who can access them. Louis Pasteur said, “Chance favors only the prepared mind.” However, the importance of access to preparation, in which mentorship plays a key role, is currently underappreciated. These so-called “serendipitous” moments that lead to novel projects, new collaborations, and career advancement are anything but chance. They are often the products of good mentorship—mentors who guide and nurture their mentees, introducing them to the right collaborators and providing targeted input at the right time. Wider spread availability of mentorship will create more opportunities for success, rather than leaving it sequestered for those with access to it. By measuring, funding and setting expectations for mentorship, institutions can leverage this critical resource for the advancement of equity in global health.

### Accessing mentorship

Beyond prioritization and funding, an important barrier to mentorship, particularly in the Global South, is the lack of sufficiently experienced mentors. However, this problem can be at least partially overcome through mentorship training as well as partnerships and peer mentoring [2]. Collaboration with more resourced partner institutions can enable distance mentoring, particularly if the partnering institution recognizes the value of mentorship broadly and not only for its own members (e.g., for promotion). Additionally, mentorship is not limited to the earlier-noted image of the senior mentor and junior mentee. Particularly when supported through mentorship training and institutional prioritization, peers can embrace mentorship principles and provide support by reviewing each other’s work and sharing of opportunities for further training and career advancement. The culture can then grow to expect and embrace mentorship in all its forms.

**Fig 1:**
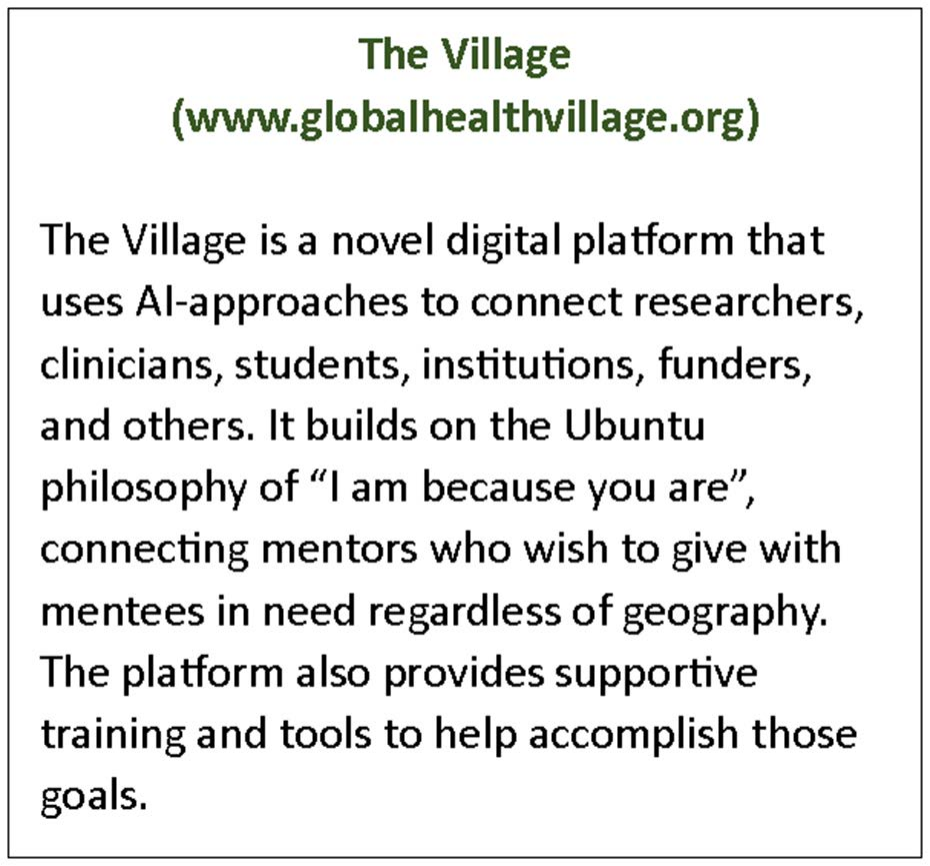
Equitable access to mentorship.

Access to mentorship can also come through the vast resources of the internet. The amount of potential talent to be empowered through high-quality mentorship throughout the world is profound, yet the connections between potential mentors and mentees are rarely made. Professional networking platforms, like LinkedIn, in theory can make these connections; however, for the equitable advancement of mentorship globally, encouragement and support of mentor-mentee matches are needed at scale. This approach is currently being developed by The Village (Fig 1), which is a novel digital platform that uses AI to connect researchers, clinicians, students, institutions, funders, and others. It builds on Ubuntu philosophy—“I am because you are”—connecting mentors who wish to give with mentees in need regardless of geography. The platform also provides supportive training and tools to help accomplish those goals for both mentors and mentees. This and similar work to scale mentorship have incredible potential for widespread impact.

### Impact from mentorship as a metric

Most people become involved in global health because they want to make the world a better place. However, the current metrics of the field—manuscripts, grants, and programmatic milestones—may or may not provide evidence for that impact. Adding mentorship as a metric with appropriate support and funding will elevate the benefits of an altruistic act to become a standard for the field, leading to profound downstream effects. Mentoring improves the quality of science and health while enhancing professional satisfaction. It also increases equity and development of homegrown solutions for increased impact, sustainability, and local autonomy. These issues are particularly important for global health. Investment in mentorship will require time and commitment to see such returns, but it will create a virtuous cycle of inspiration and fulfillment.

## Conclusion

To conclude, we advocate for the development and implementation of mentorship metrics to improve evaluation and promotion of impact in academic and career development, especially in global health. For example, institutions can set annual goals such as creating a certain number of mentoring relationships aligned with programmatic goals as well as a proportionate number of successful projects. Alternatively, programs can aim to train a certain number of mentors with the curricula identified in Table 2. These accomplishments could provide tangible markers of progress. Moreover, launching mentorship awards (e.g., through institutions and funders in global health) could celebrate outstanding mentorship efforts, inspire broader participation, and promote the culture of mentorship. We encourage institutions to implement or enhance their mentorship programs and join platforms like The Village to foster a global mentorship ecosystem. Achieving these goals will require dedicated funding to integrate mentoring as an essential responsibility of global health researchers and practitioners and makei it a core performance indicator. Finally, we emphasize the importance of promoting mentorship relationships that flow from Global South mentors to Global North mentees, as these relationships exemplify the equity, reciprocity, and shared learning we aspire to achieve in global health. In short, high-quality mentorship will help ensure that we indeed make the differences we seek in global health.
